# Genomic Mutation Profile of Primary Gastrointestinal Diffuse Large B-Cell Lymphoma

**DOI:** 10.3389/fonc.2021.622648

**Published:** 2021-03-05

**Authors:** Peifeng Li, Jia Chai, Zi Chen, Yang Liu, Jie Wei, Yixiong Liu, Danhui Zhao, Jing Ma, Kaijing Wang, Xia Li, Yang Shao, Li Gong, Wei Zhang, Shuangping Guo, Qingguo Yan, Mingyang Li, Linni Fan, Zhe Wang

**Affiliations:** ^1^State Key Laboratory of Cancer Biology, Department of Pathology, Xijing Hospital and School of Basic Medicine, Fourth Military Medical University, Xi'an, China; ^2^Department of Pathology, The 960th Hospital of the Chinese People's Liberation Army, Jinan, China; ^3^Division of Cellular and Molecular Pathology, Department of Pathology, University of Cambridge, Cambridge, United Kingdom; ^4^Department of Oral Mucosa, Affiliated Stomatological Hospital, Fourth Military Medical University, Xi'an, China; ^5^Nanjing Geneseeq Technology Inc., School of Public Health, Nanjing Medical University, Nanjing, China; ^6^Department of Pathology, Tangdu Hospital, Fourth Military Medical University, Xi'an, China

**Keywords:** diffuse large B-cell lymphoma, DLBCL, gastrointestinal tract, whole-exome sequencing, genetic mutation

## Abstract

Primary gastrointestinal diffuse large B-cell lymphoma (GI-DLBCL) is the most common gastrointestinal lymphoma, but its genetic features are poorly understood. We performed whole-exome sequencing of 25 primary tumor samples from patients with GI-DLBCL and 23 matched normal tissue samples. Oncogenic mutations were screened, and the correlations between genetic mutations and clinicopathological characteristics were analyzed. Twenty-five patients with GI-DLBCL were enrolled in the genetic mutation analysis with a median of 184 (range 79–382) protein-altering variants per patient. We identified recurrent oncogenic mutations in GI-DLBCL, including those in *TP53, MUC16, B2M, CCND3, HIST1H1C, NEB*, and *ID3*. Compared with nodal DLBCL, GI-DLBCL exhibited an increased mutation frequency of *TP53* and reduced mutation frequencies of *PIM1, CREBBP, BCL2, KMT2D*, and *EZH2*. Moreover, GI-DLBCL exhibited fewer *MYD88* and *CD79B* mutations than DLBCL in the testis and central nervous system. GI-DLBCLs with *HLA-B, MEF2A, RHOA*, and *NAV3* mutations exhibited a tendency toward a high proliferation index. *MUC16* and *ETV6* mutations often occurred in tumors with early clinical staging. Our data provide a comprehensive understanding of the landscape of mutations in a small subset of GI-DLBCLs. The genetic mutation profiles of GI-DLBCL differ from those of nodal DLBCL and DLBCL in immune-privileged sites. The different mutated genes are related to the NF-κB and JAK-STAT pathways, and the different pathogenetic mechanisms leading to the development of DLBCL may be influenced by the tissue microenvironment. Differences in genetic alterations might influence the clinicopathological characteristics of GI-DLBCL.

## Introduction

Diffuse large B-cell lymphoma (DLBCL) is the most common aggressive non-Hodgkin lymphoma. It is subdivided into multiple morphological variants given its complex and diverse histological characteristics. Based on gene expression profiling, DLBCL is classified into the following two molecular subtypes: activated B-cell-like (ABC) subtype and germinal center B-cell-like (GCB) subtype (1). Therefore, DLBCL is a clinically, morphologically, immunologically, and genetically heterogeneous diagnostic category (2, 3). Its molecular genetic characteristics have been thoroughly studied with the advent of next-generation sequencing (4). Chromosomal copy-number aberrations, gene rearrangements, and mutations promote the occurrence and progression of DLBCL (2, 5).

DLBCL occurs not only in the lymph nodes but also in many extranodal sites (6–8). Approximately 40% of DLBCLs are manifested at primary extranodal sites, and the gastrointestinal tract is the most common extranodal site (1, 7). Previous studies have shown that genetic mutations in gastrointestinal DLBCL (GI-DLBCL) are not identical to those in non-gastrointestinal DLBCL. GI-DLBCL has a significantly reduced frequency of *CD79B* and *MYD88* mutations compared with nodal DLBCL and extranodal DLBCL of immune-privileged sites (9–11). These targeted analyses of select alterations suggest that the pathogenesis of GI-DLBCL might differ from that of non-gastrointestinal DLBCL. However, comprehensive research on the genetic mutation landscape specifically focused on GI-DLBCL remains lacking, and the correlation between its genetic mutation and clinicopathological features is poorly understood.

In this study, we examined 25 primary tumor samples from patients with GI-DLBCL using whole-exome sequencing and analyzed their genetic mutation characteristics. The screened oncogenic mutations were compared with mutation data from other DLBCL cases. In addition, the correlations between gene mutations and clinicopathological features were analyzed. We attempted to elucidate the genomic mutation profile of GI-DLBCL and its clinicopathological significance and provide insights into the mechanisms of lymphomagenesis in GI-DLBCL.

## Materials and Methods

### Patient Cohort

The institutional ethical committee of Xijing Hospital and Tangdu Hospital approved this study, and written informed consent was obtained from each patient. Twenty-five patients were selected from the clinical database of both hospitals based on a recorded pathological diagnosis of GI-DLBCL. All patients met the criteria for primary gastrointestinal lymphoma as defined by Lewin et al. (12). Given that the combined therapy of surgery and chemotherapy is superior to other treatment strategies for gastrointestinal lymphoma (13–15), many patients with GI-DLBCL underwent surgical treatment in both hospitals. Twenty-three surgical resection specimens and two biopsy specimens with a histopathological diagnosis of GI-DLBCL were used in this study, and all specimens were reviewed by two senior hematopathologists (mostly Z.W., S.G., or W.Z.) according to the criteria published in the 2017 WHO classification (1). All patients had sufficient formalin-fixed, paraffin-embedded tumor tissues, and 23 of 25 patients had available corresponding normal tissue samples. Patient clinicopathological information was collected, and evaluation and scoring of various features were strictly performed in accordance with corresponding international standards (16).

### Whole-Exome Sequencing

DNA extraction of formalin-fixed, paraffin-embedded tissues was performed as described previously (17). Whole-exome sequencing was performed using a targeted capture approach with the Agilent SureSelect Human All Exon Kit (Santa Clara, CA, USA) followed by massively parallel sequencing of enriched fragments on the Illumina HiSeq Platform (Geneseeq Technology Inc., Nanjing China). Tumor and normal DNA samples were sequenced to an average depth of 101.2× and 42.2× in targeted exonic regions, respectively. All tumor specimens had an average sequencing depth of the target region >60× and coverage of the target region >90% at 20× (Supplementary Figure 1A). All reads were aligned to the human reference genome (hg19) using BWA version 0.7.12, and duplicate reads were removed using the Picard tool (http://broadinstitute.github.io/picard/).

### Identification of Somatic Single Nucleotide Variants (SNVs) and Indels

Variants were identified by UnifiedGenotyper based on hg19 and annotated with dbSNP version 144 (https://www.ncbi.nlm.nih.gov/SNP/). Variants in tumor samples were filtered against pooled normal samples, and synonymous SNVs, and intronic variants were removed. Variants identified in the 1,000 Genomes database (https://www.1000genomes.org/) with a frequency >1% (unless they were in the Catalog of Somatic Mutations in Cancer (COSMIC) database) or in the Exome Aggregation Consortium (http://exac.broadinstitute.org/) with a frequency >0.1% were discarded from the analysis. Variants with an alternate allele depth <2 and a frequency <5% were also excluded. In addition, SNVs and indels were filtered to remove benign changes predicted by ≥5 of the nine predictive software programs, including Polyphen2_HDIV, Polyphen2_HVAR, MutationTaster, Mutation Assessor, FATHMM, Radial SVM, LR, SIFT, and LRT.

### Mutational Landscapes of Common DLBCLs

To more clearly define the commonalities and differences between GI-DLBCL and non-gastrointestinal or nodal DLBCL, we analyzed the mutational landscapes of common DLBCL with COSMIC and cBioPortal (https://www.cbioportal.org/). In COSMIC, a filtered file on DLBCL was acquired from genome-wide screens (including whole-exome sequencing), and coding point mutations of 256 samples (https://cancer.sanger.ac.uk/cosmic/) were analyzed, including one case of primary breast tumor, one case in soft tissue, two cases in the abdomen, two cases in the spleen, one case in the tonsil, 23 cases in lymph nodes, and 226 unspecified cases. In cBioPortal, 1,446 profiled samples of DLBCL with mutation data were collected, including seven cohorts of DLBCL patients [DFCI (Dana-Farber Cancer Institute), Nat Med 2018 (*n* = 135); Broad, PNAS 2012 (*n* = 58); Duke, Cell 2017 (*n* = 1,001); TCGA (The Cancer Genome Atlas), PanCancer Atlas (*n* = 48); BCGSC (BC Cancer Agency's Genome Sciences Center), Blood 2013 (*n* = 53); MD Anderson Cancer Center (*n* = 148); and BCGSC, Nature 2011 (*n* = 10)]. Unfortunately, we could not obtain the primary sites of these 1,446 tumors. Furthermore, Zehir et al. (18) detected mutations in 74 patients with DLBCL using MSK-IMPACT (Memorial Sloan Kettering Cancer Center-Integrated Mutation Profiling of Actionable Cancer Targets), which revealed mutations in 410 or 341 genes. There were 35 cases of nodular DLBCL and 5 cases of GI-DLBCL in MSK-IMPACT. The mutational differences between GI-DLBCL and nodal DLBCL in MSK-IMPACT were analyzed to clarify the unique mutation profile of GI-DLBCL.

### Statistical Analysis

Statistical analysis was performed using IBM-SPSS Statistics version 23 (IBM, Armonk, NY, USA). Logistic regression was used to analyze the correlations between gene mutations and clinicopathological characteristics. The Mann–Whitney *U*-test was employed to analyze the relationship between the number of mutations or mutated genes and clinicopathological characteristics. Genetic mutation differences between the data and those obtained from COSMIC, cBioPortal and MSK-IMPACT were investigated using the Pearson χ^2^ test. Statistical significance was defined as a *P* < 0.05.

## Results

### Clinicopathological Characteristics of GI-DLBCL

**Figure 1:**
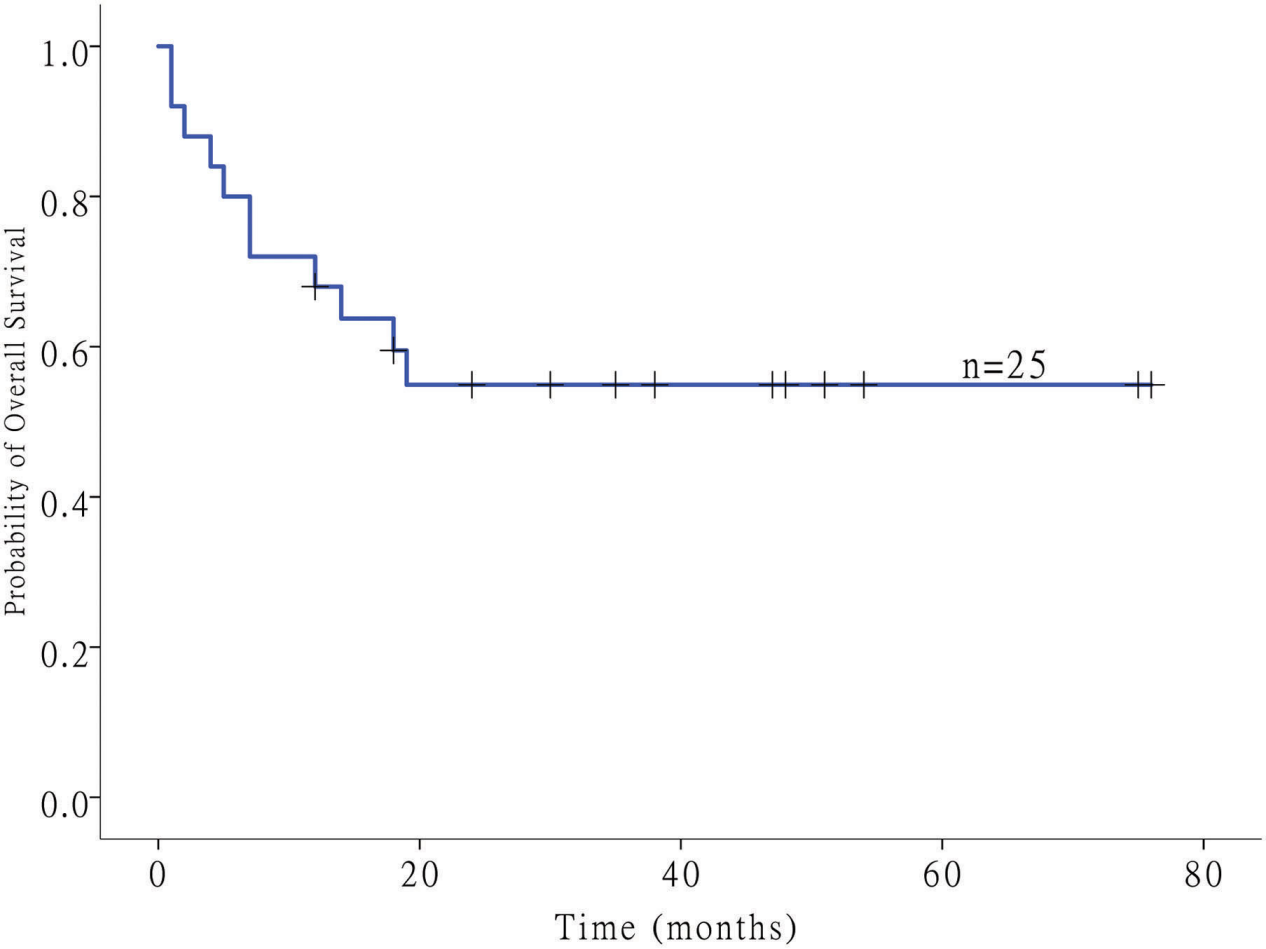
The Kaplan–Meier survival curve showed the overall survival in 25 patients with GI-DLBCL.

Twenty-five patients with GI-DLBCL were enrolled in our study. Twelve (48.0%) of 25 patients were female. The age at the time of diagnosis ranged from 21 to 83 years, and the average age was 48.7 ± 15.3 years (median: 49 years). Tumors were present in the stomach of 12 patients, all of whom received total gastrectomy. Three patients with small intestine tumors underwent partial small intestine resection, and an additional 10 patients with colon DLBCL underwent partial colectomy. All patients received postoperative CHOP-based chemotherapy (rituximab, cyclophosphamide, doxorubicin, vincristine and prednisone). Of the 12 gastric cases, four cases showed *Helicobacter pylori* infection but lacked MALT (extranodal marginal zone lymphoma of mucosa-associated lymphoid tissue) characteristics, including dense infiltration of centrocyte-like cells in the lamina propria and typical lymphoepithelial lesions. Tumors were classified into GCB (*n* = 6) and non-GCB (*n* = 19) molecular subtypes based on immunohistochemical features. No statistically significant differences in patient age (<50 or ≥50 years), sex or tumor location (stomach or intestine) were noted between GCB-DLBCL and non-GCB-DLBCL in this study. *MYC* and *BCL2* status was evaluated in 15 and 12 cases of DLBCL by fluorescence *in situ* hybridization, respectively. *MYC* translocations were observed only in one case, while *BCL2* translocation was not observed. Most samples exhibited a high Ki-67 index (>90% in 5 samples, 60~90% in 18 samples, and <60% in two samples). The clinical stage was stage I in 4 patients (16%), stage II in 4 patients (16%), stage III in 12 patients (48%), and stage IV in 5 patients (20%). The follow-up duration ranged from 1 to 76 months (median: 19 months) (Figure 1). The clinicopathological characteristics of the GI-DLBCL patients are summarized in Table 1 and Supplementary Table 1.

**Table 1:** Clinicopathological characteristics of 25 patients with GI-DLBCL.

**Parameter**	**Patients (%)**
**Age (years)**	
<50	13 (52.0%)
≥50	12 (48.0%)
**Sex**	
Female	12 (48.0%)
Male	13 (52.0%)
**Tumor site**	
Stomach	12 (48.0%)
Small intestine	3 (12.0%)
Colorectum	10 (40.0%)
**Ann Arbor stage**	
I–II	8 (32.0%)
III–IV	17 (68.0%)
**B symptoms**	
Yes	14 (56.0%)
No	11 (44.0%)
**ECOG performance status**	
<2	12 (48.0%)
≥2	13 (52.0%)
**IPI risk group**	
0–1	11 (44.0%)
2–5	14 (56.0%)
**Molecular subtype**	
GCB	6 (24.0%)
Non-GCB	19 (76.0%)

*GI-DLBCL, gastrointestinal diffuse large B-cell lymphoma; ECOG, Eastern Cooperative Oncology Group; IPI, international prognostic index; GCB, germinal center B-cell*.

### Mutational Landscapes of GI-DLBCL Identified by Whole-Exome Sequencing

We performed whole-exome sequencing of 25 patient-derived tumor specimens, and 23 patients had matched normal DNA. A total of 4,999 exonic mutations were identified in 25 patients with GI-DLBCL. Of these, 3,562 were missense variants, 332 were stop-gain mutations, 42 were stop-loss mutations, 164 were non-frameshift deletions, 148 were non-frameshift insertions, 78 were frameshift deletions, 64 were frameshift insertions, 389 were unknown-function mutations, and 220 were splice site mutations. Mutations with the number of base alterations equal to the sequencing depth and mutations with an unknown function were removed, resulting in a median of 184 (range 79–382) protein-altering variants (SNVs and small indels) per patient (Supplementary Figure 1B).

### Oncogenic Driver Genes in GI-DLBCL

**Figure 2:**
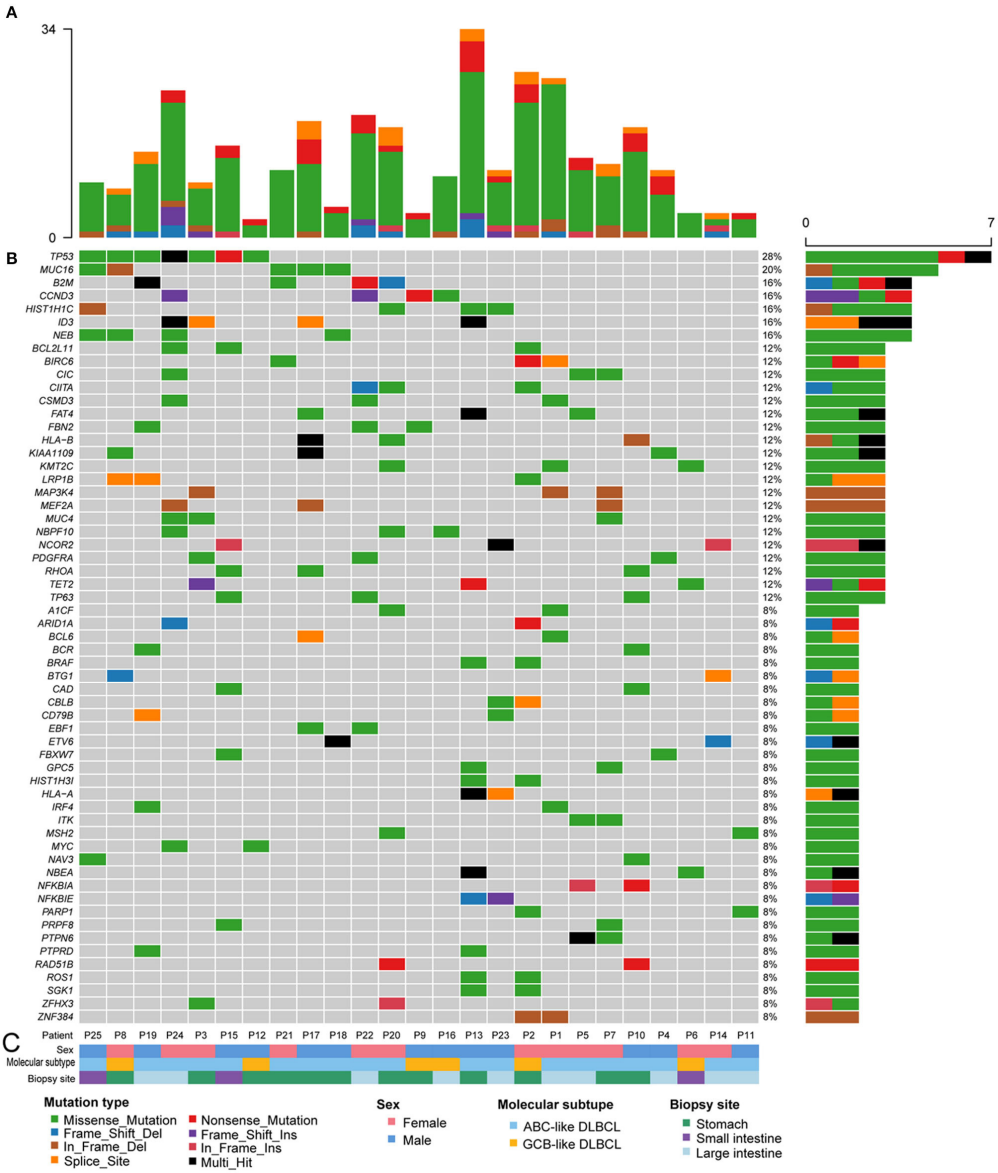
Recurrently mutated oncogenes in 25 patients with GI-DLBCL. **(A)** The absolute number of oncogenic mutations in each patient. **(B)** Fifty-nine recurrently and significantly mutated genes constitute the individual rows and are sorted according to their mutational frequencies. The heatmap represents individual mutations in 25 patient samples color-coded by the mutation type. Right: histogram shows the number of mutations in each gene. Percentages represent the fraction of tumors with at least one mutation in the specified gene. **(C)** Tracks at the bottom of the plot provide information on sex, the molecular subtype and biopsy site. Bottom: the mutation type, sex, molecular subtype, and biopsy site are color-coded as indicated in the legend.

**Figure 3:**
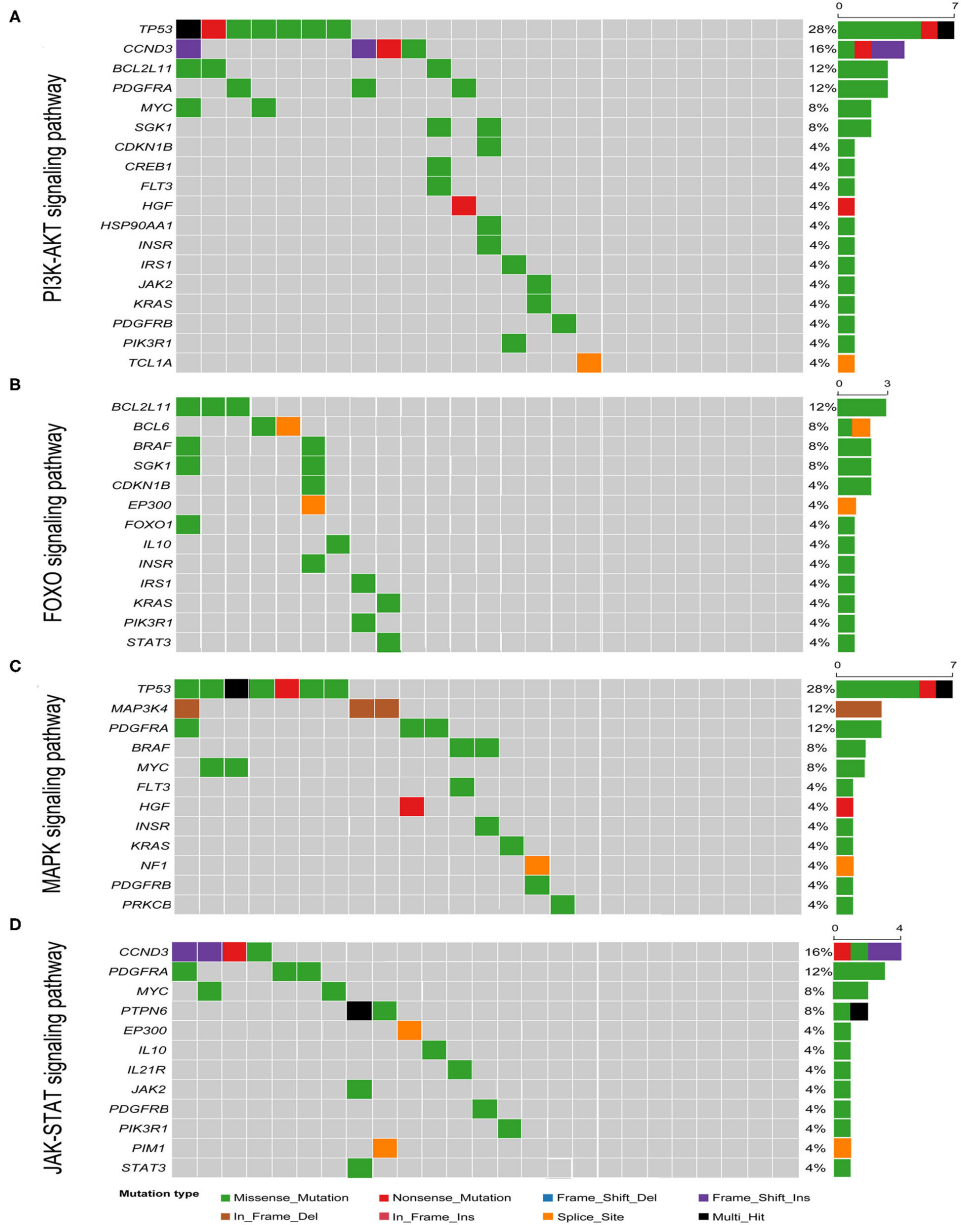
Heatmap of pathways most commonly affected by genetic mutations in GI-DLBCL. **(A)** PI3K-AKT signaling pathway; **(B)** FOXO signaling pathway; **(C)** MAPK signaling pathway; **(D)** JAK-STAT signaling pathway. Right: histogram shows the number of mutations in each gene. Percentages represent the fraction of tumors with at least one mutation in the specified gene.

Known pathogenic genes associated with human tumors were determined with the aid of COSMIC, MDG125 (19), SMG127 (20), CDG291 (21), and a recently published reference (18). A total of 333 mutations of various mutation types were identified in 189 genes. There were 242 missense mutations, 33 non-sense mutations, 21 splice site mutations, 12 in-frame deletions, 6 in-frame insertions, 12 frameshift deletions, and 7 frameshift insertions. The number of mutated genes ranged from 3 to 28 per patient with a median of 11 mutated genes (Figure 2 and Supplementary Table 2). There was no obvious difference in the gene mutations between gastric DLBCL and intestinal DLBCL. KEGG pathway analysis revealed that many of the mutated genes are involved in cancer, signal transduction, infectious disease, and immune system pathways. Crucial signal transduction pathways included the PI3K-AKT, FOXO, MAPK, and JAK-STAT signaling pathways (Figure 3). The mutated genes potentially promote tumor cell proliferation and evasion of apoptosis through cascade reactions. No obvious differential pathways were noted between GCB-DLBCL and non-GCB-DLBCL.

Among the 189 mutated genes, 59 recurrent genes were identified as candidate oncogenes in 25 patients with GI-DLBCL, and the mutational frequencies, mutation types and clinical/molecular features are displayed in Figure 2. At least two recurrent oncogenic mutations were detected in each tumor. These gene-sets prominently included members of the PI3K-AKT (*TP53, CCND3, BCL2L11, PDGFRA, MYC*, and *SGK1*), T cell receptor (*RHOA, CBLB, ITK, NFKBIA, NFKBIE*, and *PTPN6*) and MAPK (*TP53, MAP3K4, PDGFRA, BRAF*, and *MYC*) signaling pathways. *TP53* (*n* = 7) was the most frequently mutated gene, and *MUC16* (*n* = 5), *B2M* (*n* = 4), *CCND3* (*n* = 4), *HIST1H1C* (*n* = 4), *NEB* (*n* = 4), and *ID3* (*n* = 4) were also commonly mutated. The mutation characteristics and possible effects on their proteins are illustrated in Supplementary Figure 2. Interestingly, *CCND3* and *ID3* typically exhibited truncating mutations, which may lead to loss of function of these proteins.

### Contrasting Mutational Landscapes to Non-gastrointestinal DLBCL

**Figure 4:**
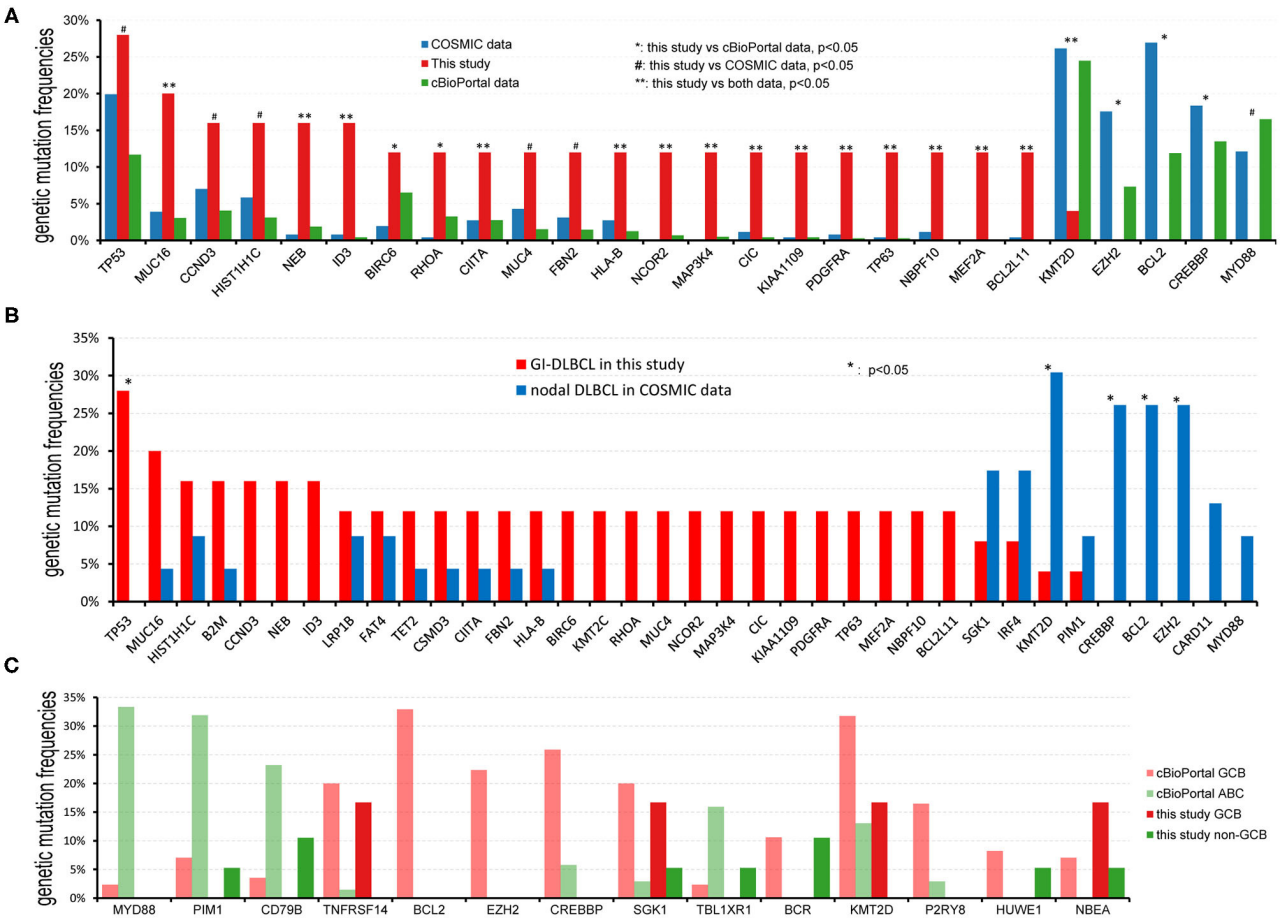
Comparison of the mutational frequencies of the most commonly mutated oncogenes in DLBCL. **(A)** Comparison of mutation frequencies between GI-DLBCL and DLBCL in cBioPortal and COSMIC. GI-DLBCL exhibited different mutation profiles compared with common DLBCL in cBioPortal and COSMIC. **(B)** Comparison of mutation frequencies between GI-DLBCL and nodal DLBCL in COSMIC. **(C)** Comparison of mutation frequencies for different oncogenic mutations between GCB-DLBCL and non-GCB-DLBCL identified in cBioPortal; no gene exhibited different mutation frequencies between GCB-DLBCL and non-GCB-DLBCL in this study.

To investigate whether genetic alterations in GI-DLBCL are similar to those in common DLBCL, the mutational frequencies of oncogenic driver genes in GI-DLBCL were compared with those in common DLBCL in cBioPortal and COSMIC (Supplementary Table 3). In cBioPortal, genetic mutations in 1,446 patients with DLBCL were analyzed. *KMT2D* (24.48%), *MYD88* (16.53%), *PIM1* (16.46%), *CREBBP* (13.49%), *BCL2* (11.89%), *TP53* (11.69%), *HIST11E* (11.07%), and *CARD11* (10.93%) exhibited mutation frequencies >10%. The *TP53* gene showed more mutations in our cohort (*P* = 0.023), but mutations in *MYD88, CREBBP, BCL2*, and *CARD11* were not found. Our study also revealed a lower mutation frequency for the *KMT2D* gene (*P* = 0.001; Figure 4A). Similar results were obtained in COSMIC. *BCL2, KMT2D, CREBBP*, and *EZH2* all exhibited high mutation frequencies in COSMIC, but few or no mutations in these genes were observed in 25 patients with GI-DLBCL (*P* < 0.05; Figure 4A). Furthermore, we analyzed the mutation differences between GI-DLBCL in this study and nodal DLBCL in COSMIC (*n* = 23). GI-DLBCL exhibited a higher mutation frequency in *TP53* than nodal DLBCL (*P* = 0.010) and a lower mutation frequency in *KMT2D* (*P* = 0.020), *BCL2* (*P* = 0.008), *CREBBP* (*P* = 0.008), and *EZH2* (*P* = 0.008; Figure 4B). Moreover, compared with nodal DLBCL in MSK-IMPACT, GI-DLBCL also exhibited a significantly lower mutation frequency of *KMT2D* (*P* = 0.003), *PIM1* (*P* = 0.035), *BCL2* (*P* = 0.016), *CREBBP* (*P* = 0.000) and *EZH2* (*P* = 0.036) in our study (Supplementary Table 3). These comparisons indicate that GI-DLBCL has a mutation profile that differs from that of non-gastrointestinal and nodal DLBCLs. GI-DLBCL exhibited an increased mutation frequency in *TP53, MUC16, CCND3, HIST1H1C, NEB*, and *ID3* and a reduced mutation frequency in *MYD88, CREBBP, BCL2, KMT2D, PIM1*, and *EZH2*.

DLBCL has GCB and ABC molecular subtypes, and these subtypes have different mutation cohorts. In cBioPortal, the mutation features of 85 patients with GCB-DLBCL were compared to those of 69 patients with ABC-DLBCL, and many oncogenic driver genes revealed different mutation frequencies (Figure 4C). GCB-DLBCL tumors harbored more mutations in *BCL2, EZH2, CREBBP, KMT2D, SGK1, BCR, NBEA, P2RY8, HUWE1*, and *TNFRSF14*, while ABC-DLBCL tumors harbored more mutations in *MYD88, PIM1, CD79B*, and *TBL1XR1*. However, the mutation frequencies and variants observed in 6 patients with GCB-DLBCL and 19 patients with non-GCB-DLBCL in our study were not different. The most likely cause of this finding is that some genetic mutations, such as those in *MYD88, CREBBP, BCL2, KMT2D, PIM1*, and *EZH2*, rarely occur in GI-DLBCL. In addition, the incidence of genetic mutations in *TNFRSF14, TBL1XR1*, and *P2RY8* was reduced in GI-DLBCL compared with those in non-gastrointestinal DLBCL; however, the differences between the two were not statistically significant (Supplementary Table 3). Furthermore, we analyzed the mutation differences between non-GCB-DLBCL in this study (*n* = 19) and ABC-DLBCL in cBioPortal (*n* = 69). *MYD88* and *PIM1* mutations were significantly reduced in non-GCB-type GI-DLBCL (*P* = 0.002 and 0.019, respectively), but mutations in *ID3, CIC, PDGFRA, MEF2A, RHOA, MAP3K4, TP63*, and *NCOR2* were increased in these GI-DLBCLs. In addition, no differences were noted in the mutation profiles of GCB-DLBCL in this study (*n* = 6) and GCB-DLBCL in cBioPortal (*n* = 85). The small number of patients in our study may be one reason for the statistical homogeneity.

### Genetic Mutations and Clinicopathological Characteristics

We analyzed the correlation between the number of genetic mutations or oncogenic mutations and clinicopathological characteristics, such as age, sex, tumor site, Ki-67 proliferation index, Eastern Cooperative Oncology Group (ECOG) score, B symptoms, International Prognostic Index (IPI), tumor stage and molecular subtype. The number of gene mutations and the number of mutant genes were not related to the abovementioned clinicopathological indicators. In addition, logistic regression analysis also revealed that patient age, sex, B symptoms, ECOG score, and tumor site were not related to oncogenic mutations. However, some mutations exhibited a tendency toward a high proliferation index (Ki-67 index >90%), such as *HLA-B* (*P* = 0.031), *MEF2A* (*P* = 0.031), *RHOA* (*P* = 0.031) and *NAV3* (*P* = 0.003). *LRPIB* mutations occurred in tumors with low IPI scores (*P* = 0.037). *MUC16* and *ETV6* mutations occurred in early-stage tumors (*P* = 0.010 and 0.032, respectively).

## Discussion

Several studies have examined the mutational landscape of DLBCL, and many recurrent genetic mutations have been detected. The most commonly mutated genes include *KMT2D, TP53, B2M, EZH2, GNA13, MEF2B, SGK1, CREBBP, CD79B*, and *MYD88* (1). In our study, GI-DLBCL exhibited high-frequency mutations in *B2M, CCND3, HIST1H1C, BIRC6, TET2, KMT2C, LRP1B, CSMD3, FAT4, RHOA, MUC4*, and *FBN2*, revealing consistent mutation frequencies and variants in common with DLBCL in cBioPortal and COSMIC. Furthermore, we rediscovered the previously discovered important drivers of DLBCL, such as *SGK1, BRAF*, and *BTG1* (22, 23); however, these genes did not exhibit high mutation frequencies. The mutations mentioned above are involved in many cellular processes and pathways, including cell growth, proliferation, migration, differentiation, metabolism, survival, apoptosis, and immunoregulation (3).

Genetic classification based on multiple analytic platforms, including mutations, translocations, and/or copy-number alterations, divides DLBCL into distinct clusters with discrete genetic signatures and different clinical characteristics (24). Chapuyg et al. (25) discovered six robust subsets (C0–C5) by applying non-negative matrix factorization consensus. Schmitz et al. (2) identified four prominent genetic subtypes termed MCD, BN2, N1, and EZB. Recurrently, seven genetic subtypes are related to distinct indolent lymphoma types, including MCD, BN2, N1, EZB, A53, ST2, and others (26). In our study, *TP53* mutations were detected in 7 cases of GI-DLBCL, and *SGK1* and *TET2* mutations were observed in four cases. Two cases had *CD79B* mutations, but no *MYD88* mutation was detected. Mutation of *EZH2, NOTCH1*, or *NOTCH2* was not detected. Simultaneously, *BCL2* translocation was not detected in 13 cases of GI-DLBCL by fluorescence *in situ* hybridization. Although GI-DLBCLs cannot be accurately classified given the incompleteness of our data, most GI-DLBCL cases in this study may have the characteristics of A53 and ST2 genetic subtypes, and a few cases were classified as MCD, EZB and N1 subtypes. However, in the study by Wright et al., the A53 and ST2 subtypes accounted for only 6.6 and 4.7% of DLBCL cases, respectively. This finding suggests that GI-DLBCL may exhibit different molecular genetic changes from other DLBCL.

The driver genes responsible for the development of gastrointestinal DLBCL differed from those of nodal or non-gastrointestinal DLBCL (27). First, *TP53* mutations demonstrated a significantly increased mutation frequency compared with that in nodal DLBCL in COSMIC and common DLBCL in cBioPortal. Similarly, Voropaeva et al. (28) reported a *TP53* mutation frequency of 16.22% in 74 patients with DLBCL, and nodal DLBCL and extranodal DLBCL exhibited mutation frequencies of 9.52 and 25.00%, respectively. Furthermore, in our study, most mutations (6/7) in *TP53* occurred in the DNA binding domain (Supplementary Figure 2), which is believed to have an adverse prognostic impact (29, 30). Second, the mutation frequencies of *MUC16, NEB, ID3, CIITA, HLB-A, NCOR2, MAP3K4, CIC, KIAA1109, PDGFRA, TP63, NBPF10, MEF2A*, and *BCL2L11* in this study were also increased compared with those in common DLBCL in cBioPortal and COSMIC. Third, mutations in the *BCL2, KMT2D, CREBBP, EZH2*, and *MYD88* genes are considered common genetic alterations in DLBCL with mutation frequencies of >10%; however, except for one mutation in the *KMT2D* gene, mutations in any of the above genes were not detected in our study. Consistent with our study, the MSK-IMPACT Clinical Sequencing Cohort reported a mutation frequency of 37.84% for *KMT2D* in 74 patients with DLBCL, but *KMT2D* mutation was not detected in 5 cases of GI-DLBCL (18). Frick et al. (9) and Nagakita et al. (31) reported that *MYD88* mutations exhibited less involvement in GI-DLBCL than in nodal DLBCL and other extranodal DLBCLs. In our previous study, the *EZH2* Y641 mutation was successfully detected in one of 94 patients with GI-DLBCL (including 33 patients with GCB-DLBCL and 61 patients with non-GCB-DLBCL) by Sanger sequencing, and the mutation frequency was significantly reduced compared with that in non-gastrointestinal DLBCL (16). Therefore, we hypothesized that the mutation profile of GI-DLBCL differed from that of nodal DLBCL and non-gastrointestinal DLBCL.

The different mutation profiles between GI-DLBCL and non-gastrointestinal DLBCL suggest different pathogenesis. In addition to mutations in the *KMT2D, MYD88, CREBBP, BCL2* and *EZH2* genes, *PIM1* and *CARD11* gene mutations were also observed at a lower frequency in GI-DLBCL than in common or nodal DLBCL (32, 33); however, the difference was not statistically significant in this study (Figure 4 and Supplementary Table 3). Some of these gene mutations are related to the NF-κB pathway (*BCL2, MYD88*, and *CARD11*) and JAK-STAT pathway (*BCL2, CREBBP*, and *PIM1*). Gene mutations leading to overactivation of the NF-κB-related pathway is an important molecular mechanism of DLBCL lymphomagenesis. Gene mutations in *CARD11, CD79A, CD79B*, and other genes activate the NF-κB pathway through the B-cell receptor signaling pathway (2, 34), and *MYD88* mutations promote IRAK4 and IRAK1 phosphorylation and activate the NF-κB pathway in an antigen-independent manner (35). Moreover, in barrier-protected tissues, such as the testis (59–61%) and central nervous system (75–94%), *MYD88* mutations are frequently detected (10, 11, 25, 36). Studies have reported missense mutations in the *CARD11* gene in 9.6% of ABC-DLBCLs (34). However, *MYD88* and *CARD11* mutations were not detected in 25 patients with GI-DLBCL. Intriguingly, although *CD79B* mutations were detected in two patients (8%) with GI-DLBCL, the frequency was significantly reduced compared with that in the testes (18.9~71.4%) (9, 11, 37) and central nervous system (31.6~61.1%) DLBCLs (36, 38, 39). Moreover, only one case had an activating mutation at p.Y197 of *CD79B* gene (Supplementary Table 2). These results are indicative of different pathogenetic mechanisms leading to the development of DLBCL, which may be influenced by the tissue microenvironment. The gastrointestinal tract has immunologically specific environments where antigen stimuli are fulminant, and the NF-κB pathway is activated by antigen stimulation in the chronic inflammatory environment. Interestingly, 4 of 12 patients with gastric DLBCL exhibited *Helicobacter pylori* infection in our study. However, in barrier-protected tissues where tumor cells experience less antigenic stimulation, NF-κB pathway activation is particularly dependent on chronic B-cell receptor signaling and Toll-like receptor signaling maintained by *CARD11, CD79B*, and *MYD88* mutations (31). In addition, many low-frequency oncogenic mutations also promote lymphomagenesis (Supplementary Table 3), and KEGG pathway analysis of the mutant genes in this study revealed that the PI3K-AKT pathway might play an important role in promoting GI-DLBCL tumor cell growth. Genetic mutations were detected in *CCND3, MYC, PDGFRA, SGK1, BCL2L11, PDGFRB*, and *PIK3R1*, all of which perform cellular functions through the PI3K-AKT pathway (2). Therefore, DLBCLs in different primary sites have various mutation characteristics due to their unique microenvironment. Primary DLBCL in immune-privileged sites exhibits high-frequency mutations in *MYD88* and *CD79B*, and nodal DLBCL exhibits high-frequency mutations in *KMT2D, PIM1, BCL2, CREBBP*, and *EZH2*. However, these mutations rarely occur in GI-DLBCL.

Some high-frequency mutations, such as mutations in *KMT2D, TP53, CD79B, CARD11, EZH2*, and *MYD88*, play important roles in lymphomagenesis (23); presumably, however, additional alterations are also indispensable for the development and characterization of DLBCL (5). We analyzed the correlation between the number of mutations and clinicopathological characteristics. Neither the number of somatic mutations nor the number of oncogenic mutations was associated with the following clinicopathological characteristics: sex, age, tumor site, molecular subtype, Ki-67 proliferation index, B symptoms, ECOG score, IPI score, and clinical stage. Similarly, Cascione et al. (40) analyzed 72 patients with MALT lymphomas by next-generation sequencing, and there was no association between the mutational status and clinical stage. However, some genetic mutations, such as those in *HLA-B, MEF2A, RHOA*, and *NAV3*, were associated with a high tumor proliferation index in this study. In addition, patients with *ETV6* mutations are associated with early clinical stages of GI-DLBCL. In DLBCL, the *ETV6* mutation is a common gene alteration, with a frequency of 3.25% and a preference for the ABC subtype (in cBioPortal), especially in primary central nervous system DLBCL (41, 42). In a recent study, the *ETV6* gene was identified as a driver gene of DLBCL (43).

## Conclusion

GI-DLBCL exhibited low genetic mutation frequencies of the *BCL2, KMT2D, CREBBP, EZH2, PIM1, MYD88*, and *CD79B* genes. The different mutated genes are related to the NF-κB and JAK-STAT pathways and suggest that the different pathogenetic mechanisms of GI-DLBCL and non-gastrointestinal DLBCL depend, in part, on the microenvironment. In addition, we also found that some genetic mutations are associated with a high tumor proliferation index and an early clinical stage. Therefore, the identification of these mutations may be useful for the prognostic prediction of GI-DLBCL. This study will help to extend the utility of cancer genome studies and accelerate the pace at which genetic findings are translated into therapeutic impacts.

## Data Availability Statement

The datasets presented in this study can be found in online repositories. The names of the repository/repositories and accession number(s) can be found below: GSA-Human (the Genome Sequence Archive for Human) [HRA000533] (https://bigd.big.ac.cn/gsa-human/browse/HRA000533).

## Ethics Statement

The studies involving human participants were reviewed and approved by Xijing Hospital Ethics Committee and Tangdu Hospital Ethics Committee. The patients/participants provided their written informed consent to participate in this study.

## Author Contributions

PL and ZW conceived and designed the study. PL, JC, YaL, JW, YiL, DZ, JM, KW, LG, and WZ performed the research. PL, ZC, XL, SG, QY, and ML performed the data analysis. PL and JC wrote the manuscript. ZW, LF, and ML reviewed and revised the manuscript. All authors contributed to the article and approved the submitted version.

## Conflict of Interest

YS was employed by the company Nanjing Geneseeq Technology Inc. The remaining authors declare that the research was conducted in the absence of any commercial or financial relationships that could be construed as a potential conflict of interest.
