# Supplementary material for: Genomic Mutation Profile of Primary Gastrointestinal Diffuse Large B-Cell Lymphoma
Source: Front Oncol. 2021 Mar 5;11:622648. doi: 10.3389/fonc.2021.622648 (PMC7973209; doi:10.3389/fonc.2021.622648)

**Supplementary Figures**

**Supplementary Figure 1** The analysis of whole-exome sequencing in 25 patients with GI-DLBCL. (A) Tumor sample sequencing percentage coverage at 10×, 20× and 30×. (B) Genetic mutation features identified by whole-exome sequencing. Each specimen exhibited different mutation patterns, and the number of mutations in each tumor ranged from 79 to 382. Left: mutation types are color-coded as indicated in the legend.

**Supplementary Figure 2** Lollipop plots with the distribution of somatic mutations on the linear protein and domains of the most affected genes in GI-DLBCL. A diagram of the relative positions of somatic mutations is shown for *TP53*, *MUC16*, *B2M*, *CCND3*, *HIST1H1C*, *ID3*, and *NEB*. Each lollipop denotes a unique mutation location, and its height represents the number of observed mutations. Colored bars indicate the individual protein domains. The type of the mutation is indicated in the key (bottom).

Supplementary Figure 1


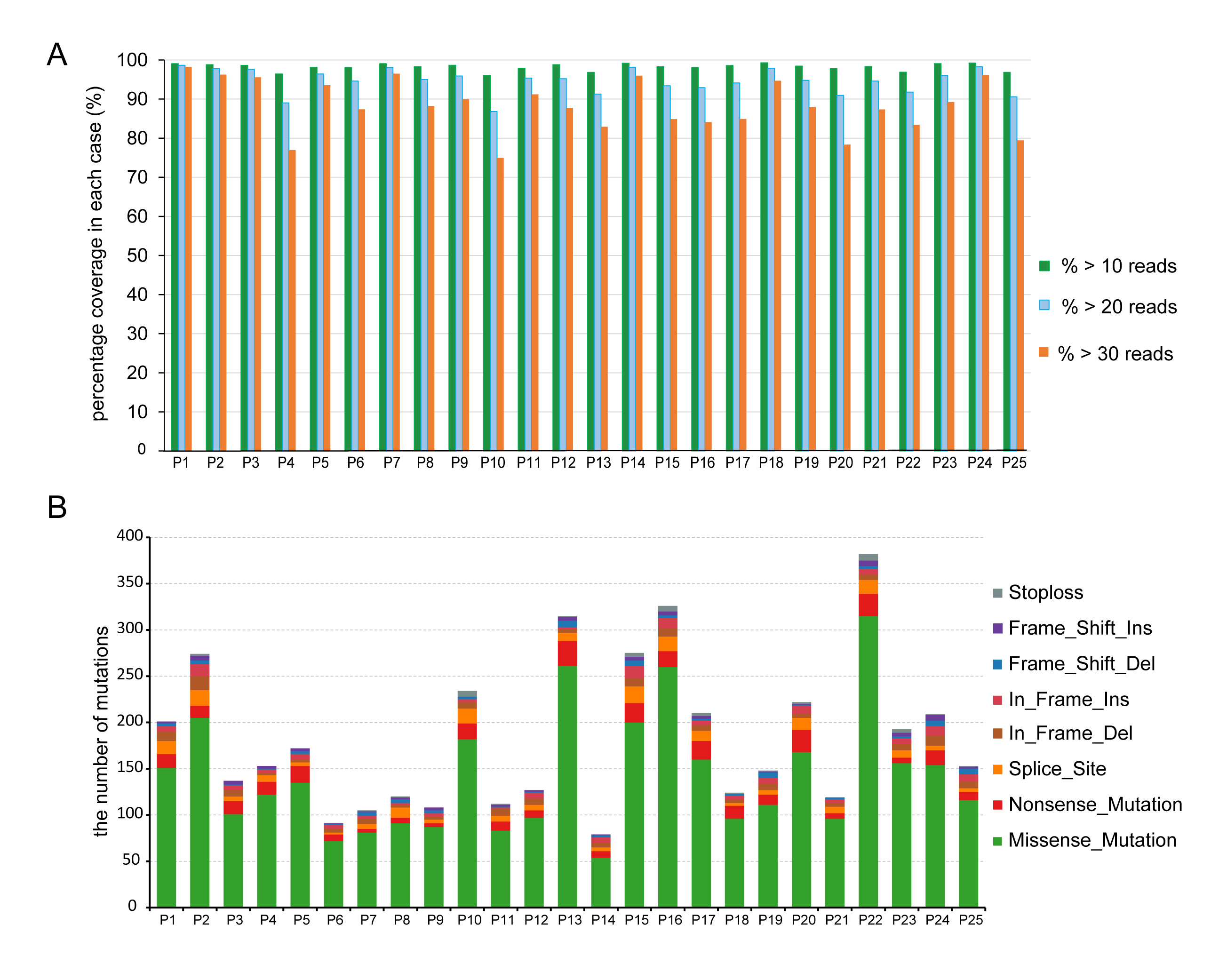


Supplementary Figure 2


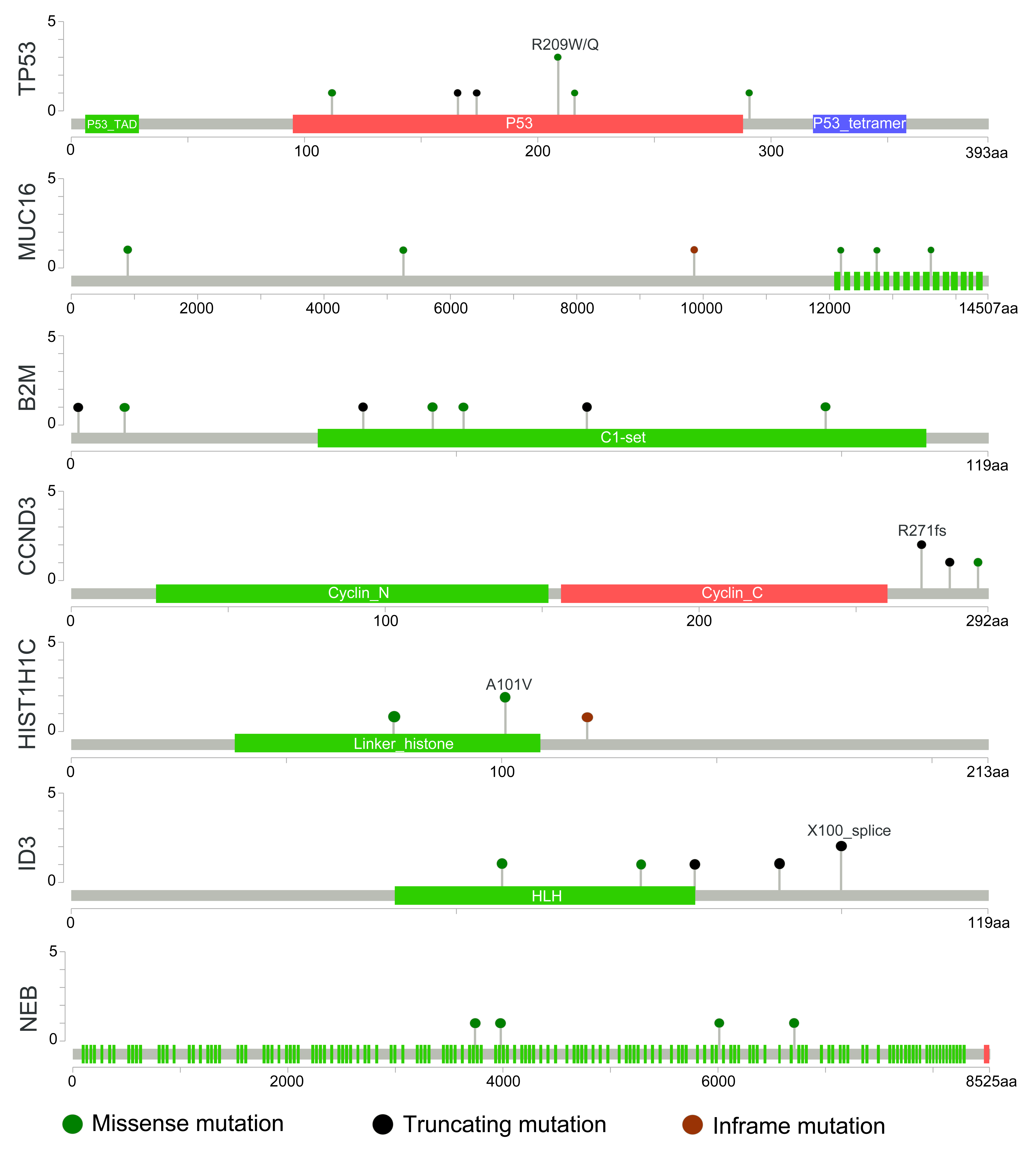

Supplement: Supplementary file 1 [file Data_Sheet_1.docx]
